# Do private providers initiate anti-tuberculosis therapy on the basis of chest radiographs? A standardised patient study in urban India

**DOI:** 10.1016/j.lansea.2023.100152

**Published:** 2023-02-02

**Authors:** Anita Svadzian, Benjamin Daniels, Giorgia Sulis, Jishnu Das, Amrita Daftary, Ada Kwan, Veena Das, Ranendra Das, Madhukar Pai

**Affiliations:** a Department of Epidemiology, Biostatistics and Occupational Health, McGill University, Montreal, QC, Canada; b McGill International TB Centre, McGill University, Montreal, QC, Canada; c McCourt School of Public Policy, Georgetown University, Washington, DC, USA; d School of Epidemiology and Public Health, Faculty of Medicine, University of Ottawa, Ottawa, ON, Canada; e Centre for Policy Research, New Delhi, India; f Dahdaleh Institute of Global Health Research, School of Global Health, York University, Toronto, ON, Canada; g Centre for the Aids Programme of Research in South Africa MRC-HIV-TB Pathogenesis and Treatment, Research Unit, Durban, South Africa; h Division of Pulmonary and Critical Care Medicine, Department of Medicine, University of California, San Francisco, CA, USA; i Department of Anthropology, Johns Hopkins University, Baltimore, USA; j Institute for Socio-Economic Research on Development and Democracy, Delhi, India; k Manipal McGill Program for Infectious Diseases, Manipal Centre for Infectious Diseases, Manipal Academy of Higher Education, Manipal, Karnataka, India

**Keywords:** Tuberculosis, Quality of care, India, Private health providers, Standardized patients, Mystery clients

## Abstract

**Background:**

The initiation of anti-tuberculosis treatment (ATT) based on results of WHO-approved microbiological diagnostics is an important marker of quality tuberculosis (TB) care. Evidence suggests that other diagnostic processes leading to treatment initiation may be preferred in high TB incidence settings. This study examines whether private providers start anti-TB therapy on the basis of chest radiography (CXR) and clinical examinations.

**Methods:**

This study uses the standardized patient (SP) methodology to generate accurate and unbiased estimates of private sector, primary care provider practice when a patient presents a standardized TB case scenario with an abnormal CXR. Using multivariate log-binomial and linear regressions with standard errors clustered at the provider level, we analyzed 795 SP visits conducted over three data collection waves from 2014 to 2020 in two Indian cities. Data were inverse-probability-weighted based on the study sampling strategy, resulting in city-wave-representative results.

**Findings:**

Amongst SPs who presented to a provider with an abnormal CXR, 25% (95% CI: 21–28%) visits resulted in ideal management, defined as the provider prescribing a microbiological test and not offering a concurrent prescription for a corticosteroid or antibiotic (including anti-TB medications). In contrast, 23% (95% CI: 19–26%) of 795 visits were prescribed anti-TB medications. Of 795 visits, 13% (95% CI: 10–16%) resulted in anti-TB treatment prescriptions/dispensation and an order for confirmatory microbiological testing.

**Interpretation:**

One in five SPs presenting with abnormal CXR were prescribed ATT by private providers. This study contributes novel insights to empiric treatment prevalence based on CXR abnormality. Further work is needed to understand how providers make trade-offs between existing diagnostic practices, new technologies, profits, clinical outcomes, and the market dynamics with laboratories.

**Funding:**

This study was funded by the 10.13039/100000865Bill & Melinda Gates Foundation (grant OPP1091843), and the Knowledge for Change Program at 10.13039/100004421The World Bank.

**Research in context**

**Evidence before this study**

In the Indian private sector, there is evidence that quality of TB care falls short of international standards in many places and is in urgent need of improvement. The evidence comes from systematic reviews on the quality of TB care or surrogates of quality indicators such as diagnostic delays, long patient pathways, analyses of TB care cascades, and newer standardized patient (SP) studies that directly measure quality of TB care. Some issues highlighted by these sources point to low rates of TB testing by private providers, low rates of referral to the national TB program, and widespread empirical management with antibiotics. Chest radiographs (CXR) are indicated as a preferred test for many private providers.

**Added value of this study**

This study is the first to make use of multiple rounds of SP data across two large urban Indian settings in order to provide insights into the prevalence of empiric treatment based on a CXR abnormality and providers’ behaviours associated with this practice.

**Implications of all the available evidence**

Our finding that one in five SPs with an abnormal CXR was prescribed anti-TB treatment by private providers is important evidence of fairly widespread empirical management of TB and might help explain the low rates of bacteriological TB diagnoses in the Indian private health sector. Such practices could potentially compromise the timely diagnosis of TB, miss drug-resistance, and result in inappropriate drug regimens. We discuss the implications of the study findings on quality of TB care more broadly while suggesting opportunities for laboratory and health systems strengthening. A more detailed qualitative inquiry into provider practices and decision-making rationale, coupled with engaging them in antimicrobial stewardship and lab strengthening measures (e.g., free or subsidized WHO recommended rapid diagnostics) are likely to have impacts on timely and appropriate management of TB and other respiratory infections.

## Introduction

An important marker of high-quality tuberculosis (TB) care is the initiation of anti-TB treatment (ATT) based on the results of WHO-approved microbiological diagnostics. Only 60% of the 4.8 million people diagnosed with pulmonary TB worldwide in 2020 were microbiologically confirmed. Remaining patients are diagnosed clinically, that is, based on signs and symptoms, and/or by abnormalities on chest radiographs (CXR) or other non-microbiological tests.^1^ Microbiological detection of TB is critical because it allows patients to be correctly diagnosed, is a prerequisite to test for drug resistance, and ensures that the most effective treatment regimen is initiated, based on the drug resistance pattern.

The WHO thus urges National TB Programs (NTPs) to make use of WHO recommended diagnostics (WRDs) that are more sensitive than smear microscopy.^2^ Recently, WHO updated its guidelines for the use of molecular assays intended as initial tests for the diagnosis of TB: amongst both adults and children in the general population with signs and symptoms of pulmonary TB or with CXR with lung abnormalities or both, Xpert MTB/RIF or Xpert Ultra (Cepheid, Sunnyvale, CA, USA) or TrueNAT MTB (Molbio Diagnostics, India) should replace smears as the initial test for pulmonary TB.^2^ In India, the NTP's current algorithm has been revised to reflect current standards, and includes the use of CXR as a screening test and the upfront use of rapid molecular tests.^3^ The NTP guidelines recommend that all efforts must be made to confirm a TB diagnosis microbiologically, and defines an CXR-based diagnosis as ‘clinically diagnosed TB.’

Some studies that have examined diagnostic and treatment patterns for TB have shown that some providers initiate treatment in the absence of bacteriologically confirmed diagnosis, referred to as empirical TB treatment.^4^^,^^5^ Drivers of empiric management of TB include, amongst other factors, a high burden of disease, difficulty in accessing sputum TB testing, easy access to antibiotics, clinical presentations suggestive of TB, excessive reliance on non-specific tests such as CXRs, and widespread antibiotic abuse.^6^ While not all diagnoses of TB can be microbiologically confirmed, it is important for healthcare providers to make a serious effort at getting a diagnostic confirmation. TB symptoms are neither sensitive nor specific, and tests such as CXR lack specificity, with the implication that an incorrect diagnosis of TB could lead to unnecessary TB treatment.^5^ Empiric treatment can potentially result in overtreatment leading to possible drug-related toxicity, added costs to patients and the health system, and a missed opportunity to correctly diagnose and treat other diseases that mimic TB.

India is the biggest consumer of antibiotics, and antibiotic abuse has worsened during the COVID-19 pandemic.^7^ Studies demonstrate that private providers in India often rely on clinical diagnosis and empiric treatment initiation.^4^^,^^5^ In India, only 16% of all private TB notifications in 2018 were bacteriologically confirmed, suggesting widespread empirical management.^8^ Prior research shows varied patterns of diagnostic algorithms employed by providers, such as clinical diagnosis without diagnostic testing or clinical diagnosis with a CXR suggestive of TB, with low utilization of microbiological tests and high rates of empirical treatment.^9^^,^^10^ Private and informal providers in these contexts decide to rely rather on their experience and clinical acumen and can also be swayed by profits.^11^ Moreover, TB drug prescription data analyses have found that the amount of TB medications sold in the private sector is two to three fold that of the number of notified patients.^12^ Despite this disjunct, it remains impossible to link this directly to the potential for overtreatment of TB since true TB burden is measured in terms of numbers of patients, not volumes of anti-TB drugs sold in the market.^13^

Given the evidence to date, it is likely that providers are initiating treatment without sufficient investigations. However, it remains unclear the extent to which empiric treatment on the basis of clinical judgement and/or abnormal chest CXR is occurring. In this study, we employed the standardized patient (SP) methodology to directly address this exact question. Building off our previous work,^10^^,^^14^, ^15^, ^16^, ^17^, ^18^, ^19^ we examine our question with a unique dataset available from a large-scale SP study, where SPs presented with abnormal CXRs to formally trained private healthcare providers in the two Indian cities of Patna and Mumbai across three data collection waves.

### Context

Our study settings are Patna and Mumbai, India. Mumbai is the relatively wealthy and cosmopolitan capital of the state of Maharashtra. As per the latest census (2011),^20^ it is home to 12 million inhabitants, with an annual per capita income of 180,000 Indian Rupees (INR) (equivalent to US$ 2440). In contrast, Patna is the capital of the state of Bihar and one of India's less developed states, with a recorded per capita income of 30,441 INR (US$ 412). The population of urban Patna is 2 million.^20^

In 2019, 106,189 and 191,294 patients with TB were officially notified in Bihar state and Maharashtra state, respectively.^3^ Within the public sector, in Bihar, 43,139 (40.6%) cases were confirmed via microbiological confirmation whereas 79,039 (41.3%) were confirmed in Maharashtra.^3^ A country-wide prevalence survey conducted between 2019 and 2021 indicated that the prevalence of microbiologically confirmed pulmonary TB among population aged ≥15 years was observed to be 327 per 100,000 (95% CI: 236–417 per 100,000) population in Patna and 161 per 100,000 (95% CI: 105–218 per 100,000) population in Mumbai.^21^ In this survey, all eligible study participants underwent symptom screening using a standard questionnaire and CXR screening; participants with respiratory symptoms suggestive of TB and/or with past history of TB and/or currently on TB treatment and/or having an abnormal CXR were eligible for sputum examination. The diagnostic algorithm in the survey highlighted the importance of using molecular tests in diagnosing TB given that 33% of cases were exclusively diagnosed based on a molecular test.^21^ Molecular tests, when combined with a diagnosis by smear and culture methods, increased the detection of TB to 78.4%. Moreover, it was found that 42.6% of the TB cases in the survey would have been missed if CXR had not been included.^21^

Data on how many cases were diagnosed empirically within the private sector of each of these two cities does not exist, though the country-wide survey indicated that 67% and 66% presented in the private sector in Bihar and Maharashtra, respectively.^21^ In 2018, only 16% of all private notifications were bacteriologically confirmed across the country.^8^ The reasons for which this happens despite the availability of microbiological diagnostic tools has yet to be properly explored in these contexts. In terms of the coverage of molecular testing across the country, as of 2020, the number of rapid molecular machines increased from 1547 modules in 2019 to 3147 in 2022. Of these, 72 rapid molecular testing platforms were housed in Bihar and 335 machines in Maharashtra.^21^

While both cities have public clinics and hospitals, it is within the largely unregulated private sector where most patients decide to seek care.^21^ It is important to note that the structure of the private sector is very different between both these cities. Informal providers in Mumbai are mainly AYUSH (Ayurveda, Yoga & Naturopathy, Unani, Siddha, and Homeopathy) practitioners, while in Patna informal providers tend to be those with other or no qualifications at all. Our previous SP work found very low rates of anti-TB drug prescription by AYUSH and informal providers (who, legally, are not allowed to prescribe ATT), while formal providers accounted for a large majority of ATT prescriptions.^18^

This present study focused solely on MBBS-trained, formal providers in each of these two cities, as they are the practitioners who have the ability to order microbiological testing or prescribe ATT, as shown in our previous work.^18^ Therefore, we examined their propensity to prescribe microbiological diagnostic tests prior to the commencement of any treatment.

## Methods

### Study design and data collection

To describe care decisions, patterns of prescription and treatment practices based on CXR abnormalities among formally trained private practitioners in Patna and Mumbai, we analyzed unique SP data collected in three rounds of data collection: round 1 for each city occurred in 2014–2015, round 2 in 2016–2018, and the final round 2019–2020.

Assessing quality of TB care, and within that, reasons for which empirical diagnoses are chosen over microbiological diagnoses, can be challenging. Generally, quality of care studies have relied on recall-based patient surveys, questionnaire surveys of knowledge, and prescription/chart analyses. Unfortunately, these methods are prone to significant biases and thus may not reflect genuine practice.^22^, ^23^, ^24^, ^25^, ^26^, ^27^ To overcome some of these issues, SPs are increasingly considered as the gold standard for measuring provider practice in low- and middle-income countries (LMICs) and settings where medical records are lacking. Compared to other methods, SP studies can provide an accurate assessment of provider practice that is free from observation bias, less vulnerable to recall bias and allows for valid quality comparisons across different types of health care providers.^25^^,^^28^ There is extensive literature documenting the validity and benefits of this approach.^14^^,^^17^^,^^29^ The SP method was validated for assessing TB quality of care in urban India.^10^^,^^16^^,^^24^^,^^27^

We followed the Strengthening the Reporting of Observational Studies in Epidemiology (STROBE) guidelines (Supplementary STROBE Checklist).

### Case presentations

Individuals recruited and trained to be SPs in each round portrayed one of two case scenarios designed by a multidisciplinary team of medical anthropologists, health economists, epidemiologists, and local clinical experts. The methods of SP recruitment and training are described elsewhere.^18^^,^^19^ “Case 1” consisted of a classic symptomatic case of presumptive TB who had had 2–3 weeks of cough and fever. “Case 2” was a presumptive TB case who had 2–3 weeks of cough and fever as well, but additionally presented with a CXR and had received broad-spectrum antibiotic treatment for one week as ordered by another provider, with no improvement. The cases are further detailed in Table 1.

**Table 1:** Standardised patient case scenario descriptions.

	Case description	Presentation of patient	Expected correct case management
**Case 1**	Classic case of presumed tuberculosis with 2–3 weeks of cough and fever	Presents with presumptive tuberculosis, for the first time, to a private health-care provider, saying “Doctor, I have cough that is not getting better and some fever too”	Recommendation for sputum testing, chest radiograph, or referral to a public DOTS center or qualified provider
**Case 2**	Classic case of presumed tuberculosis in a patient who has had 2–3 weeks of cough and fever. The patient has taken a broad-spectrum antibiotic (amoxicillin) given by another health-care provider for 1 week with no improvement. He also carries an abnormal chest CXR suggestive of tuberculosis	Presents after an initial, failed (empirical) treatment for symptoms with broad-spectrum antibiotics and a diagnostic chest CXR, saying “I have cough and fever which is not getting better. I went to a doctor and took the medicines he gave me and have also had an CXR done.” The chest CXR and blister pack for the antibiotics are shown if the provider asks	Recommendation for sputum testing, chest radiograph, or referral to a public DOTS center or qualified provider

DOTS = directly observed therapy, short-course; CXR = chest radiograph. Reproduced under (CC BY 4.0)^18^.

More specifically, for case 2, the SP carried a digital CXR dated within the last 10 days with evidence of abnormalities, and a blister pack of amoxicillin with them. The antibiotic was only shown when the provider asks for it. The SP began the interaction by saying: “Doctor, I have had cough and fever. It is not getting better, even though I went to a doctor and took medicines also.”

The same CXR film (Supplement figures) and same radiologist report was used for all rounds, both in Patna and Mumbai; male and female SPs had corresponding sex-specific reports. The letterhead on which the report was prepared as per the location deployed. The CXR film itself showed an ill-defined patchy parenchymal opacity with the accompanying report stating that the abnormality was possibly due to “Pulmonary Koch's” (as TB is often reported on radiology reports in India). We decided to include both the report and the film since we did not expect all practitioners to be able to read the film, for the sake of uniform and consistent interpretation of radiological findings. We wanted to eliminate any possibility that a provider may incorrectly read the CXR film and conclude that there were no abnormalities present. Both the film itself and the accompanying report contained information that could have been indicative of other respiratory conditions (e.g., bacterial pneumonia) which would require further clinical investigation and microbiological testing to diagnose.

### Weighting

Based on our sampling strategy (detailed in Supplement) the city-level estimates of the behavioural characteristics in Mumbai and Patna were extrapolates from the sampling frame to the full population of private healthcare providers in each city. These are further detailed in S1 Text 3. Table 2 details the weights employed for Case 2 presentations, by type of provider and city with S1-Table 2 in the Supplementary materials detailing case 1 weighting as well.

**Table 2:** Sampling and weighting distributions for case 2 within formal providers surveyed.

	Weighting Group				
**Round**		Patna Formal Non-PPIA	Patna Formal PPIA	Mumbai Formal Non-PPIA	Mumbai Formal PPIA
**Round 1** **(2014–2015)**	n	70	28	69	53
	weight	0.01274152	0.00386048	0.01387193	0.00080824
**Round 2** **(2016–2018)**	n	112	45	76	60
	weight	0.00796345	0.00240208	0.01259425	0.00071395
**Round 3** **(2019–2020)**	n	94	41	90	57
	weight	0.00948837	0.00263643	0.01063515	0.00075152

PPIA = public-private interface agency; SP = standardized patient.

### Outcome definitions

We benchmarked our main outcome, expected correct case management, on Indian Standards for TB Care.^30^ We focused primarily on referral and diagnostic behaviours regardless of provision of any unnecessary medication. Based on national guidelines, the recommendation is to order a microbiological test or refer the patient for further management, rather than dispense any further medications or Group 1 ATT, specifically. Since we measured ordering a test or referring the SP, rather than ordering a test only, our definition of correct case management is stricter than those utilized in previous SP studies assessing quality of TB care.^29^

We operationalized management of case 2 using three binary outcome definitions. First, we defined an ideal standard of care as a (1) prescription for any microbiological test (sputum smear, Xpert MTB/RIF, or another molecular TB test (e.g., TrueNAT) or culture) and no concurrent prescription or dispensation of a steroid or any antibiotic (more specifically of quinolones or anti-TB medications). The following two definitions of empiric management were of progressively conservative nature; (2) a prescription for ATT was dispensed/prescribed, and (3) a prescription for anti-TB medication and an additional simultaneous prescription indicated for confirmatory microbiological testing, either via sputum smear microscopy, Xpert MTB/RIF, or culture. These three definitions are summarized in Table 3.

**Table 3:** Definitions of empiric treatment practices for tb and proportions by case type.

	Referral for sputum smear	Referral Xpert MTB/RIF or another molecular TB test	Referral for Culture/DST	Corticosteroid	Quinolone	Any Antibiotic	Group 1 anti-TB Medication (isoniazid, rifampicin, ethambutol, pyrazinamide)
**Definition 1:** **Ideal management**	Yes	Yes	Yes	No	No	No	No
**Definition 2:** **Empiric treatment with ATT**	No	No	No	–	–	–	Yes
**Definition 3:** **Empiric treatment with ATT with concurrent microbiological testing**	Yes	Yes	Yes	–	–	–	Yes

### Identification of medicines

Medicines dispensed or prescribed to the SPs were coded into pre-determined categories of interest (e.g., corticosteroids, antibiotics). To assess drug use, all labelled medicines prescribed by the pharmacies were digitized and stored and then coded by two co-authors with expertise in TB (AS) and infectious diseases (GS). The authors were blinded from any provider-identifying details, and they identified and categorized medicines as steroids, anti-TB drugs, quinolones, or other broad-spectrum antibiotics under maker-checker procedure, whereby dual-approval was needed by two separate people for each coding. Quinolone antibiotics were defined as Anatomical Therapeutic Chemical (ATC) codes beginning with J01M, and corticosteroids, both inhaled and systemic, defined as ATC codes beginning with H02, R01, or R03.^31^ ATT was defined as the dispensation of any of the four Group 1 drugs, i.e., first-line oral anti-TB drugs: isoniazid, rifampicin, ethambutol or pyrazinamide. The category “other antibiotic” included all antibiotics other than Group 1 ATT drugs and quinolones. Discrepancies in categorization between coders were resolved by consensus. Finally, while loose or unlabeled pills (Supplement figure) were dispensed in some interactions, no further attempts were made to identify them as it was not feasible to perform chemical drug assays at scale.

### Statistical analysis

We reported proportions for outcomes of interest, namely our 3 articulations of empiric treatment practices amongst other clinical management factors, and population mean estimates computed using inverse probability weighting^32^ with corresponding 95% CIs. The weights were calculated such that each of the 96 city–case–round combinations contributed equally to overall estimates and corresponded to the sampling framework of private-sector providers listed from the mapping exercise in both cities.

We also presented findings over time (from round 1 to round 3 of the survey). To look at time trends between rounds, we conducted weighted pair-wise t-tests and linear regressions.

In addition to using these weights to estimate population likelihoods, we used them to calculate adjusted weighted prevalence ratios (aPRs) in a multivariate model by using log-binomial regression comparing variation in factors associated with treatment based on abnormal CXR, by city, and clustering at the provider level. These methods are preferred to logistic regression estimation to directly estimate PRs from cross-sectional studies since odds ratios overestimate the PR, particularly when the outcome is not rare.^33^^,^^34^ The associations between predictors of interest and practices will be presented as a PR as a more interpretable alternative to the odds ratio. We take the perspective that a quality outcome in each interaction reflects a combination of inputs that vary at provider, patient, and facility levels. We presented both univariate and multivariate models for factors of interest, with the multiple regression framework. The model is specified in the Supplement.

All estimates clustered standard errors at the provider level and were inverse-probability-weighted based on our sampling strategy to arrive at city-round-representative interpretations of our outcome measures. Reported estimates represent the expected likelihood of the outcome occurring if a provider were chosen at random from the population of providers within each respective city and within each round's time period.

### Variables of interest

The regressions comprised the following binary or dummy predictors: clinical examination performed (yes vs. no); invasive treatment offered (yes vs. no); medical history taken (yes vs. no); study site (Patna (ref) vs. Mumbai); patients waiting in office (yes vs. no); round of data collection (round 1 (ref) vs. round 2 vs. round 3); treatment counselling performed (yes vs. no). We presented these as a univariate regression individually, and a full multivariate model with all co-variates.

All data analyses were performed with Stata 15 (Stata, College Station, TX).

### Ethics

Ethical approvals for this study were granted by the McGill University Health Centre in Montreal, Canada (REB No. 14-137-BMB) and the Institute for Socioeconomic Research on Development and Democracy in Delhi, India.^10^ Ethics committees approved a waiver from obtaining informed consent from providers in Patna and Mumbai under the Government of Canada Panel on Research Ethics, as well as a study by Rhodes and colleagues (2012) on ethical aspects of standardized patient studies commissioned by the US Department of Health and Human Services.^35^ The study specific rationale of waiver is detailed elsewhere.^18^ All individuals who participated as standardised patients were hired as staff and trained to protect themselves from any harmful medical interventions, such as avoiding injections, invasive tests, or consuming any drugs.

### Role of funding source

The funders had no role in study design, data collection and analysis, decision to publish, or preparation of the manuscript.

## Results

Amongst Case 2 interactions, that is SPs who presented an abnormal CXR, 25% (95% CI: 21–28%), adjusted for weighting, resulted in ideal management, where the provider prescribed a microbiological test (sputum smear, Xpert MTB/RIF, or culture) and did not offer a concurrent prescription for a steroid or antibiotic (more specifically of quinolone or anti-TB medication) [Table 4]. Of all Case 2 SP-provider interactions, 23% (95% CI: 19–26%) ended with prescription or dispensation of ATT. A total of 13% (95% CI: 10–16%) of interactions resulted in a prescription/dispensation of ATT plus a prescription for confirmatory microbiological testing, either via sputum smear microscopy, Xpert MTB/RIF, or culture [Table 4].

**Table 4:** Percentage of interactions resulting in the outcome of interest, defined in three different ways, weighted via inverse probability.

	n/N	Percentage	95% CI
**Definition 1:** ***Ideal management***	210/795	25%	21–28%
**Definition 2:** ***Empiric management with ATT***	182/795	23%	19–26%
**Definition 3:** ***Empiric management with ATT with concurrent microbiological testing***	140/795	13%	10–16%

### Diagnostic flow

**Fig. 1:**
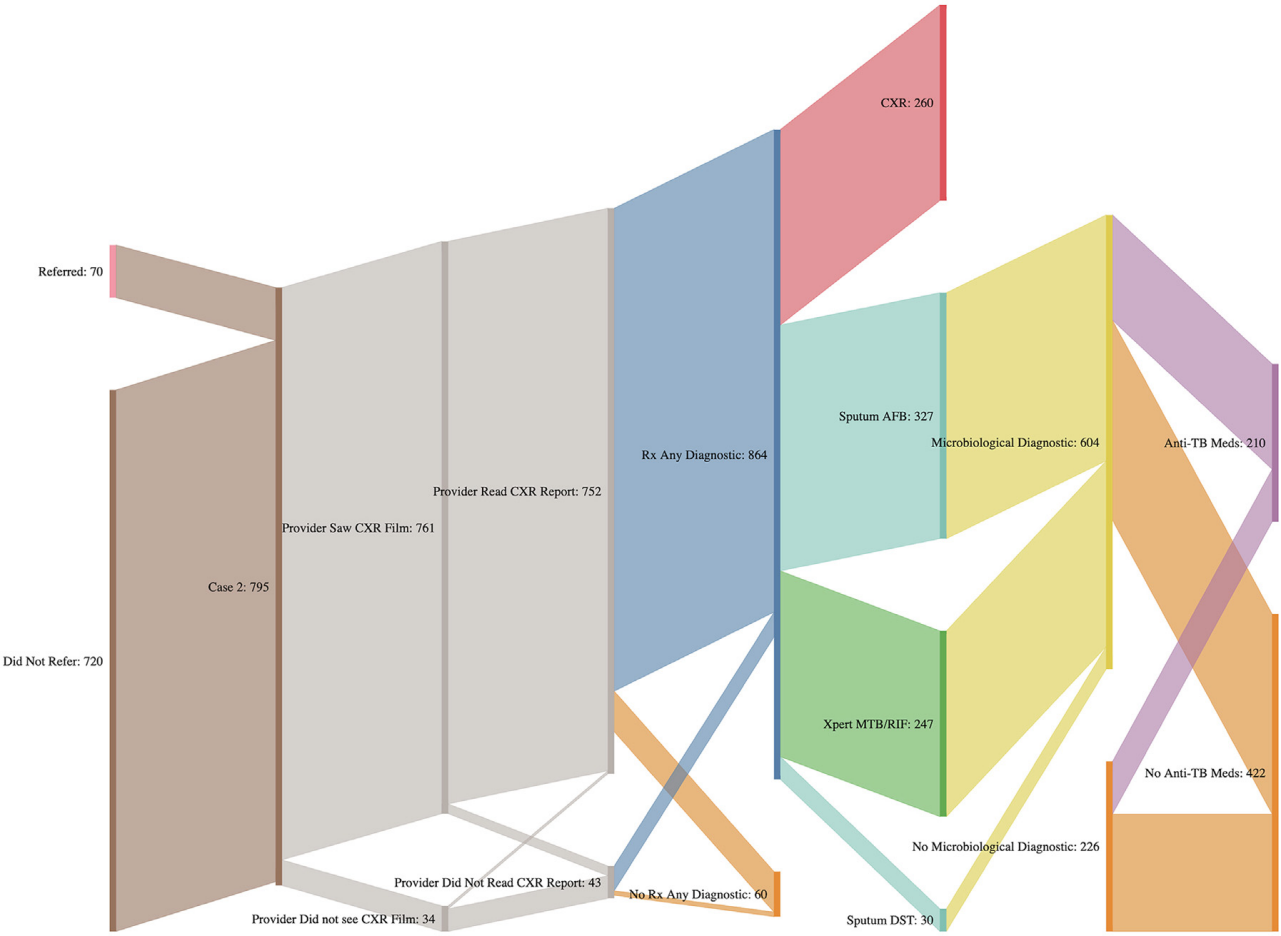
Diagnostic care cascade of patients presenting with an abnormal CXR (Case 2) and the subsequent diagnostic algorithm by which they travel through, with the dispensation (or not) of ATT regimens they are dispensed as the endpoint. These represent raw counts. CXR = chest radiography; DST = drug-susceptibility testing; AFB = acid-fast bacillus.

When we look at a diagnostic care cascade for Case 2 (Fig. 1), we see that a vast majority [761 of 795 (96%; 95% CI: 95–98%)] of providers looked at the CXR film, while 752 of 795 (95%; 95% CI: 93–97%) read the CXR report itself. A total of 686 of 795 (83%; 95% CI: 80–86%) of interactions resulted in any diagnostic test being ordered, and 266 of 794 (32%; 95% CI: 28–36%) interactions resulted in a new CXR being ordered, even though the patient would have presented with an abnormal film and a report. Moreover, 332 of 792 (43%; 95% CI: 39–48%) sputum smears, 255 of 794 (22%; 95% CI: 19–25%) Xpert MTB/RIFs and 30 of 791 (4%; 95% CI: 2–6%) cultures were prescribed. Of the 474 of 795 (53%; 95% CI: 49–57%) microbiological tests ordered, 210 of 632 (30%; 95% CI: 25–34%) interactions also prescribed or dispensed ATT medications. Only 70 of 790 (12%; 95% CI: 9–15%) interactions resulted in a referral. It is important to note that all proportions are weighted and that any inconsistencies in denominators is a reflection of many patterns being observed simultaneously for any given interaction. For example, during an SP visit, it is entirely possible that a provider prescribed both a CXR and a sputum smear- resulting in an excess of diagnostics vs. Case 2 subjects.

### Comparison of case 2 vs. case 1

**Fig. 2:**
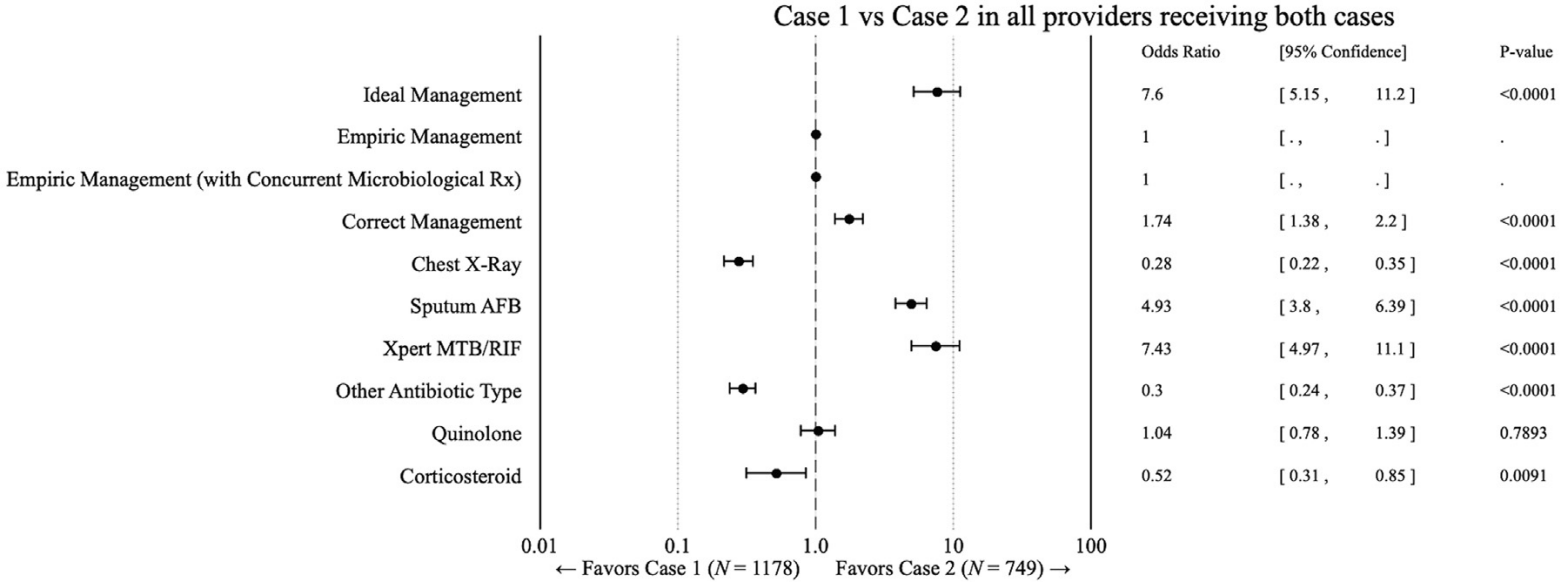
Quality of care differences between SP case scenarios presenting to the same provider calculated in a linear regression model, with standard errors clustered at the provider level. Case 1 = 1178 interactions; case 2 = 749 interactions.

Providers’ behaviours differed upon the addition of a CXR on patient presentation; when comparing providers who saw both case 1 and case 2 (n = 1178 & n = 749, respectively (Fig. 2)), the case 2 SPs carrying the CXR report resulted in increased odds of ideal management (OR 7.6; 955 CI: 5.15–11.2; p < 0.001) as well as higher odds of prescribing sputum smear (OR 4.93; 95% CI 3.80–6.39; p < 0.0001) and Xpert MTB/RIF (OR 7.43; 95% CI 4.97–11.1; p < 0.0001) and a lower likelihood of CXR ordering (OR 0.28; 95% CI 0.22–0.35; p < 0.0001), as compared to case 1. Medicines were very frequently prescribed or dispensed in both cities, and we did not penalize the use of additional or unnecessary medications against the provision of correct management. Notwithstanding, providers were less likely to order a steroid (OR 0.52; 95% CI 0.31–0.85; p = 0.0091) or other antibiotic (not quinolone or ATT) (OR 0.3; 95% CI 0.24–0.37; p < 0.0001). No statistically significant association were estimated for empiric management (across both definitions) since only 1 of 1818 case 1 interactions resulted in the prescription/dispensation of an ATT.

### Management outcomes over time

**Fig. 3:**
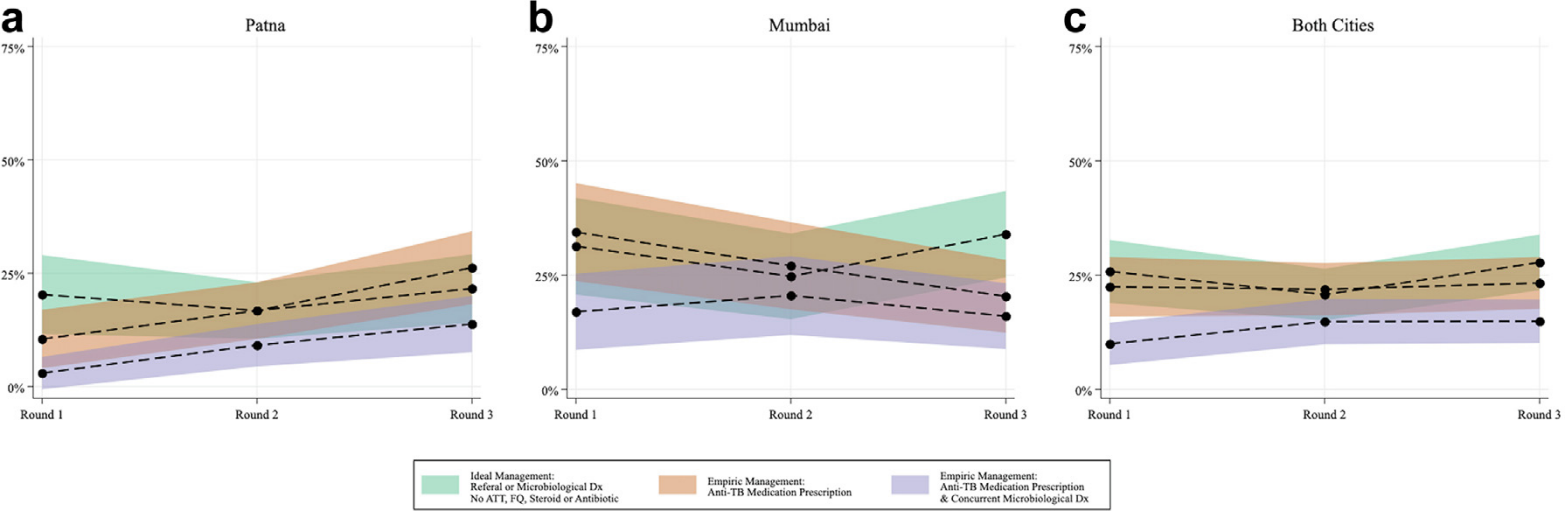
Weighted changes in empiric treatment practices by round of data collection and city, (a) Patna, (b) Mumbai, (c) both cities. Round 1: 2014–2015, round 2: 2016–2018, round 3: 2019–2020.

Considering these outcomes over time (Fig. 3), the prevalence of ideal management in Mumbai changed from 32 of 122 (31%; 95% CI: 21–42%) of visits during the first round to 30 of 136 (25%; 95% CI: 15–34%) in the second round of data collection, to 44 of 147 (34%; 95% CI: 24–43%) in the final round of data collection. Empiric treatment of presumptive TB with ATT decreased from 54 of 122 (34%; 95% CI: 24–45%) interactions at round 1, to 46 of 136 (27%; 95% CI: 17–37%) in round 2 and 34 of 147 (20%; 95% CI: 12–28%) at endline data collection, with this decreasing linear trend being statistically significant (Slope Change: −0.07, 95%CI: −0.134-(−0.0064)). Concurrent prescription of a microbiological diagnostic in addition to ATT changed over time from 32 of 122 (17%; 95% CI: 9–25%) of interactions at round 1, then subsequently 38 of 136 (21%; 95% CI: 12–29%), and finally, 29 of 147 (16%; 95% CI: 9–23%).

In Patna, ideal management was seen in 18 of 98 (20%; 95% CI: 11–29%) of interactions at round 1, 28 of 157 (17%; 95% CI: 10–23%) and 30 of 135 (22%; 95% CI: 14–29%) at the second and final round of data collection, respectively. Empiric management of case 2 was observed in 11 of 98 (10%; 95% CI: 4–17%) interactions to begin and increased to 28 of 157 (17%; 95% CI: 10–23%) and 37 of 135 (26%; 95% CI: 18–34%); here an increasing, linear trend over time was observed (Slope Change = 0.078, 95%CI: 0.028–0.129). The prescription/dispensation of ATT with a script for a simultaneous diagnostic test was observed in 3 of 98 (3%; 95% CI: 1–7%) interactions and then 17 of 157 (9%; 95% CI: 4–14%) and 21 of 135 (14%; 95% CI: 7–20%) in later rounds; this upward trend was statistically significant over time (Slope Change = 0.054, 95%CI: 0.018–0.090).

### Diagnostic practices over time

**Fig. 4:**
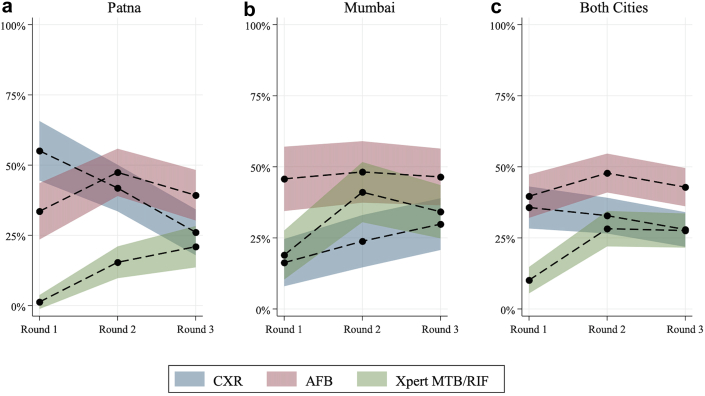
Weighted changes in diagnostic patterns by round of data collection for Case 2 and city, (a) Patna, (b) Mumbai, (c) both cities. Round 1: 2014–2015, Round 2: 2016–2018, Round 3: 2019–2020.

Looking at trends over time in diagnostic practices in both cities (Fig. 4), in Mumbai there was an increase in recommendation for repeat CXR in case 2, first observed in 24 of 122 (16%; 95% CI: 8–25%) interactions at round 1, then 35 of 136 (24%; 95% CI: 15–33%) and finally, 51 of 147 (30%; 95% CI: 20–39%). Despite the SP presenting with an existing CXR, this increase in CXR prescriptions by providers is statistically significant over time (β = 0.066, 95% CI: 0.023–0.129). Smear prescriptions stayed relatively similar over time: 49 of 122 (46%; 95% CI: 34–57%) interactions in round 1, (48%; 95% CI: 37–59%) of interactions in round 2, and (46%; 95% CI: 36–56%) of interactions in round 3. Xpert MTB/RIF diagnostic saw an initial increase from 41 of 122 (19%; 95% CI: 10–28%) of interactions at round 1 to 77 of 136 (41%; 95% CI: 30–52%) in the second round of the survey; it then decreased to 70 of 147 (35%; 95% CI: 25–44%) of interactions in the final round.

Patna saw decreased repeat CXR recommendations over time, from 53 of 98 (55%; 95% CI: 44–66%) to 70 of 157 (42%; 95% CI: 33–50%) and 33 of 134 (26%; 95% CI: 18–34%) interactions at study end; these changes were significant over time (Slope Change = −0.145, 95%CI: −0.213–(−0.077)). Sputum smear microscopy prescriptions were seen in 34 of 98 (34%; 95% CI: 23–44%) interactions initially, then increased to 77 of 157 (47%; 95% CI: 39–56%), before decreasing to 54 of 134 (39%; 95% CI: 30–48%). Xpert MTB/RIF was only ordered for 1 of 98 (1%; 95% CI: 1–4%) interactions at round 1 and increased over time, from 34 of 157 (15%; 95% CI: 10–21%) in round 2 to 32 of 134 (21%; 95% CI: 14–28%) in the final round; this increase of uptake of Xpert MTB/RIF was significant over time (Slope Change = 0.0985, 95%CI: 0.0590–0.138).

### Factors associated with management practices of case 2

**Fig. 5:**
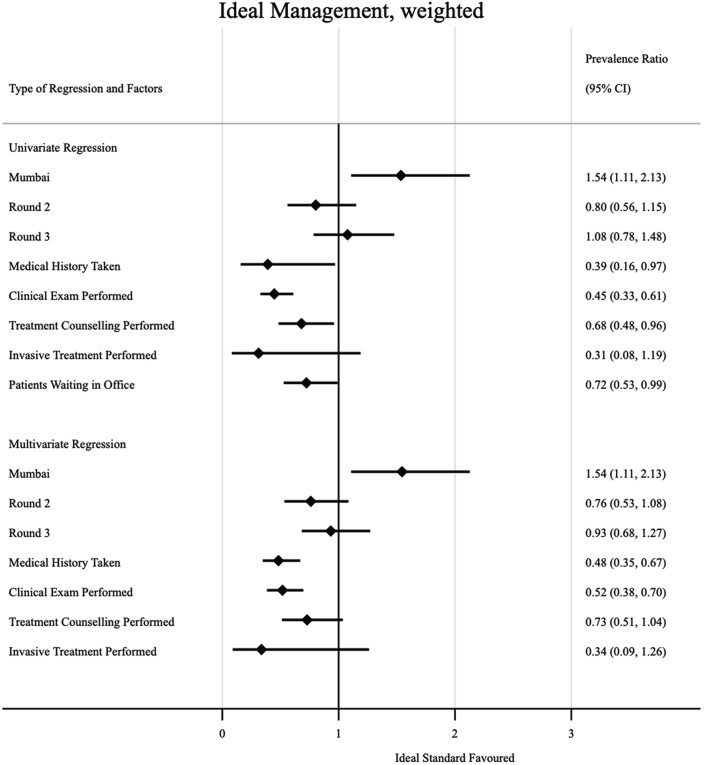
Factors associated with ideal management amongst formal providers. Prevalence ratios (PRs) were calculated via prevalence ratios (PRs) by using log-binomial regression; standard errors were clustered at the provider level, and inverse-probability-weighted based on our sampling strategy to arrive at city-round-representative interpretations of our outcome measures; the adjusted prevalence ratios (aPR) in the multivariate model adjusted for all variables included in the univariate regression.

**Fig. 6:**
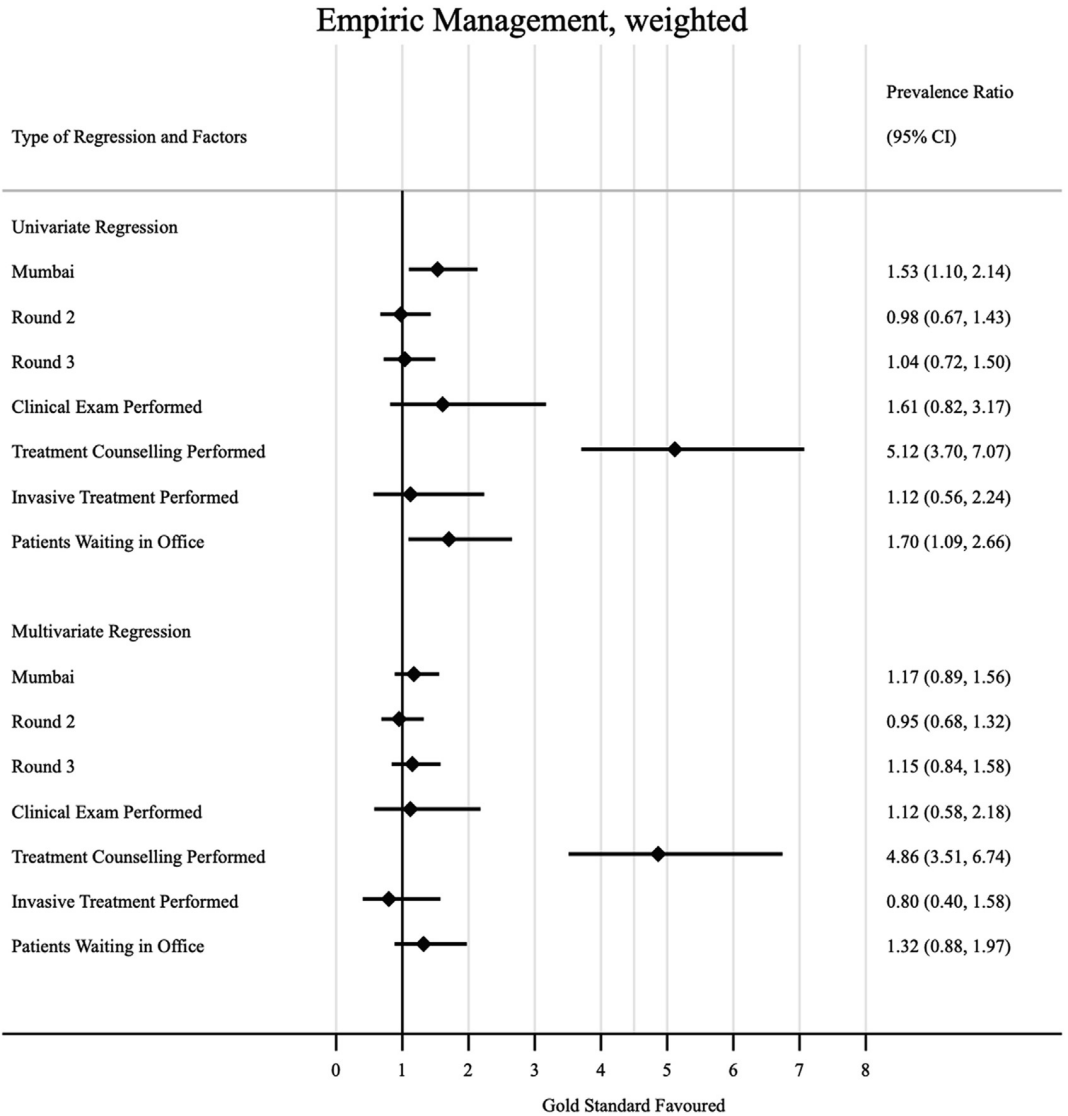
Factors associated with empiric management with ATT amongst formal providers. prevalence ratios (PRs) were calculated via prevalence ratios (PRs) by using log-binomial regression; standard errors were clustered at the provider level, and inverse-probability-weighted based on our sampling strategy to arrive at city-round-representative interpretations of our outcome measures; the adjusted prevalence ratios (aPR) in the multivariate model adjusted for all variables included in the univariate regression.

**Fig. 7:**
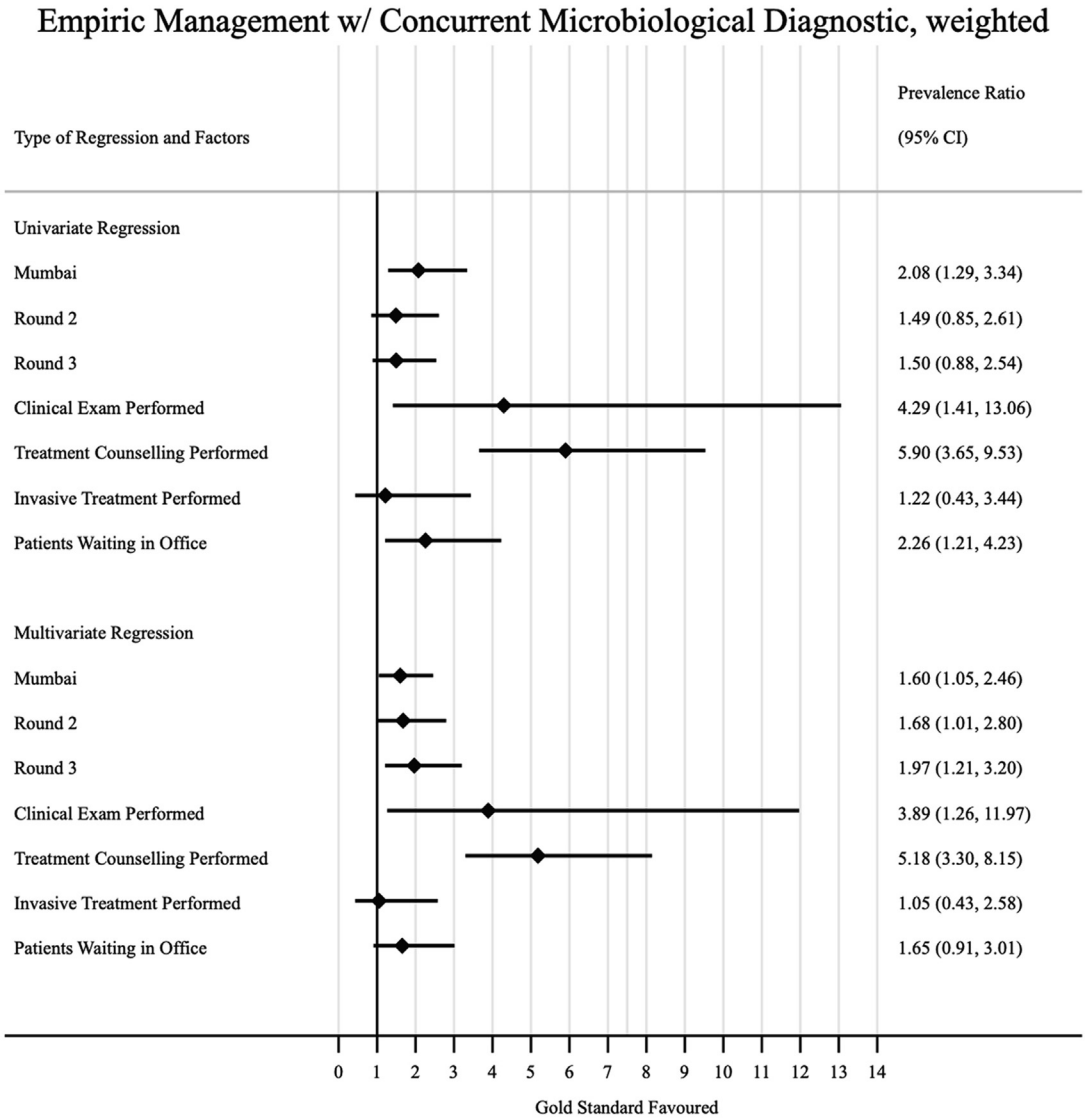
Factors associated with empiric management with concurrent microbiological diagnostic amongst formal providers. prevalence ratios (PRs) were calculated via prevalence ratios (PRs) by using log-binomial regression; standard errors were clustered at the provider level, and inverse-probability-weighted based on our sampling strategy to arrive at city-round-representative interpretations of our outcome measures; the adjusted prevalence ratios (aPR) in the multivariate model adjusted for all variables included in the univariate regression.

Some factors were associated with increased prevalence of ideal management of a case of presumptive TB with an abnormal CXR in the multivariate analysis (Fig. 5); interactions in Mumbai were more likely to result in ideal management compared to those that took place in Patna (weighted aPR 1.54, 95% CI: 1.11–2.13). The provider performing a clinical exam and taking the SP's medical history were behaviours less likely to be associated with non-ideal management (weighted aPR 0.52, 95% CI: 0.38–0.70), and (weighted aPR 0.48, 95% CI: 0.35–0.67), respectively. In Fig. 6, we see that treatment counseling was associated with empiric management of the case with ATT (weighted aPR 5.12, 95% CI: 3.70–7.07). When we also considered concurrent prescription of a microbiological diagnostic test with dispensation of ATT (Fig. 7), we see that clinical examination (weighted aPR 3.89, 95% CI: 1.26–11.97) and treatment counseling (weighted aPR 5.18, 95% CI: 3.30–8.15) were both associated with the outcome in the multivariate model.

## Discussion

This study adds to a growing body of evidence regarding practices around empiric management of TB. We explore these patterns of decision-making in the context of an abnormal CXR presentation, and also discuss the implications of the study findings on quality of TB care more broadly while suggesting opportunities for laboratory and health systems strengthening.^36^

Despite case 2 patients presenting with evidence of an ambiguous respiratory infection, without a definite indication for TB specifically (no cavitation for instance) and with proof of an ineffective course of antibiotics, antibiotics and anti-TB medications were prescribed to one in five SPs in both Patna and Mumbai. Globally, approximately 40% of reported TB cases are treated empirically,^1^ and the proportion is likely even larger in the Indian private sector, with upwards of 80% likely empirically managed in 2018 in the private healthcare sector.^8^ Yet, it remains challenging to define how much empiric treatment is too much and to target an appropriate amount of empiric treatment against an ideal standard. It is also unclear at what point the risks and costs of potential overtreatment outweigh the benefits of empiric treatment for some patients.^37^

Several issues intervene on a seamless TB diagnostic pathway. First, we know that culture, the gold standard in microbiological diagnosis, has imperfect sensitivity as a reference standard.^38^ Second, there is a general limitation in the ability of current bacteriological tools to efficiently diagnose cases with subclinical and incipient forms of TB.^39^ Finally, we are unable to know exactly what the prevalence of culture-negative pulmonary TB is within any given population and thus anticipate how much empiric treatment is truly justifiable. In patients without HIV coinfection, culture-negative TB is likely an early disease state on the continuum between *Mycobacterium tuberculosis* infection and active disease, which could advance to culture-positive disease if left untreated with ATT. Patients with negative sputum cultures may have culture-negative TB disease that would benefit from empiric treatment, and the judgment of experienced clinicians is a valuable tool for recognizing high-risk, sputum negative TB presentations.^40^, ^41^, ^42^, ^43^ In one particular study where more than 12,000 individuals were tested for TB with a highly sensitive assay in a community setting, universal testing identified both highly symptomatic individuals with high bacillary burdens (37%) and people with trace-positive sputum (63%). Within the latter group, cultures were mainly negative but patients presented with features that distinguished them from the TB-negative controls used in the study's design.^44^

A better understanding of the clinical presentation of culture-negative pulmonary TB could help clarify in which clinical scenarios initiating empiric treatment is truly appropriate.^45^ Given the approval of short course regimens, quick diagnoses are being increasingly valued and sought, to move patients through the TB care cascade.^46^^,^^47^ The risk of deferring treatment in patients with risk factors for mortality or poor follow-up, might prompt some physicians into action. However, it seems that most clinically diagnosed, culture-negative patients were found to be less sick than the typical TB case based on symptoms and inflammatory markers; treatment could have been deferred in these patients without grave consequences.^35^ In addition, empiric TB treatment of people living with HIV is associated with reduced benefits on mortality.^37^^,^^48^

Our findings are very much in line with ethnographic and qualitative research studies suggesting that multiple pressures drive empirical practices. These include the use of medications as diagnostic tools, a desire to provide rapid symptom relief to patients, providers’ uncertainty about the accuracy of available TB tests and apprehension of loss of clients in private practice.^5^^,^^11^^,^^49^ The widespread empirical use of antibiotics in India has been demonstrated in other SP studies as well.^50^ In our study, interactions where SPs were asked to return for more medicines were more likely to receive treatment prior to microbiological diagnosis, suggesting empiric antibiotic therapy may have been used as a diagnostic tool in this context. The ordering of non-specific treatments prior to microbiological diagnosis, coupled with multiple visits and providers seen, results in diagnostic delays.^8^ Our study showed that this behaviour was generally avoided when providers put in more effort, and interactions were subject to a more thorough clinical examination, whether via physical exam or more detailed history taking.

The behaviours observed may also suggest that providers might be wary of reliance on microbiological diagnosis as a tool in isolation. Prior evidence suggests that physicians also gravitate towards a multi-pronged approach to diagnostics, using clinical judgement in conjunction with various modern diagnostics, to provide a diagnosis.^5^^,^^11^^,^^49^^,^^51^ Descriptive analyses of these combinations suggested that providers may have used microbiological tests as an “add-on” test rather than as “replacement”. Xpert has been widely regarded as a game changer in the global fight against TB, but its impact on case finding and management in high-burden countries is likely to be constrained by low adoption by private providers, or by empirical treatment.^6^ Existing evidence regarding its impact is primarily from the public sector, and studies examining the integration of Xpert into clinical decision-making are limited.^52^ Compared to smear microscopy, this study found that Xpert increased the number and proportion of microbiologically confirmed TB case notifications and reduced the delay in treatment initiation. However, it did not significantly increase overall case notifications and did not have a significant impact on patient-relevant health outcomes such as mortality, TB-related morbidity, and successful treatment completion.^52^ These mixed findings are believed to be driven by high levels of empirical treatment, which may be partially reduced by a wider uptake of Xpert testing.^52^, ^53^, ^54^ Inability to do microscopy or Xpert (as in sputum-scarce patients), substandard clinical training, and high likelihood of one-off encounters with patients might also drive the initiation of empirical treatment.^6^^,^^55^^,^^56^ As health systems transition to assays with higher sensitivity, such as Xpert Ultra MTB/RIF, clinicians may benefit from guidance on how to adapt their diagnostic intuition accordingly and recalibrate their own threshold for empiric treatment.^37^

While usage of Xpert MTB/RIF does not require the deployment of highly skilled technicians, there could be fears that the accessibility of the test or delays in turnaround times would induce loss of the patient prior to diagnosis. Pre-treatment loss to follow-up in the Xpert MTB/RIF context can be due to high staff turnover, inconsistent and delayed specimen transport to Xpert MTB/RIF testing sites, time delays in obtaining results, high costs of the test, and the inability to track and follow-up patients with positive TB test results.^49^^,^^57^^,^^58^ Since these tests were almost always conducted offsite, even if test turn-around times were just a few hours, patients were often asked to return for test results and further managed in the following days, a practice that can lead to loss to follow-up.^51^^,^^59^ High cost of Xpert MTB/RIF in the private health sector might put off patients who cannot afford them, and they might never return to the providers who ordered the test.^60^ There is also the issue of practical obstacles which limit full reliance on Xpert technology despite the low skill level required to perform the test,^61^ namely that maintenance issues may force health facilities to send specimens to distant labs and cause several-day delays; such delays would increase a physicians’ propensity to choose empiric treatment for high-risk patients.^37^ In our study, we observed an overall increase in Xpert MTB/RIF utilization over time, though it remains unclear if this is associated with empiric management. While it has been shown that Xpert MTB/RIF leads to more bacteriologic confirmation among patients treated for TB,^53^ the effect of Xpert MTB/RIF on the accuracy of empirical diagnosis remains more elusive.^6^ As clinicians grow more comfortable with using Xpert MTB/RIF to rule out TB, there is some evidence that empiric treatment declines over time.^62^

Despite presenting with an abnormal CXR, many SPs were referred for repeat tests. Research in this setting suggests private providers have extensive preferred referral pathways for their patients, and relationships with their preferred labs. Another possible explanation is that private providers often have close relationships with laboratories and pharmacies, and these are based on financial incentives and relationships built over time.^49^ Thus, providers may ask for tests to be done at their preferred laboratories, including labs attached to their own clinics or hospitals, even if patients have reports from other labs.

Thus, decisions around empirical treatment of TB must be contextualized within the health market, where providers want to be able to provide care to their patients with the tools most readily accessible to them, rather than one in which the providers are blamed for mismanagement. Making WHO-endorsed tests more affordable in the private market, linking privately managed patients with free diagnostic testing in the public sector, laboratory network strengthening, and building confidence between providers and laboratories is integral to the practice of favouring microbiological diagnosis over empirical treatment alone. In Mumbai and Patna, Public Private Interface Agency programs have attempted to (i) make available high quality diagnostic tests to private providers through incentives, subsidies and eventually free of cost; (ii) provide free TB drugs and adherence support mechanisms to TB patients to maximise treatment completion; and (iii) facilitate reporting of TB patients to India's National Tuberculosis Elimination Programme. Following the success of these initiatives, private sector engagement has subsequently seen massive expansion across the country, accompanied by an increase in domestic and donor funding.^63^ Due in part to these efforts, the private sector contribution to TB notifications grew from 7% in 2014 to 28% in 2019.^3^ Such progress represents crucial steps in achieving comprehensive coverage of private providers in India.^64^

We observed that fewer providers treated with ATT prior to microbiological diagnosis in Mumbai compared to Patna. This may be partly due to the fact that Mumbai has a high prevalence of drug resistance^65^^,^^66^; Mumbai is known to have a high proportion of multidrug-resistant (MDR)-TB cases with quinolone resistance.^67^^,^^68^ Mumbai contributed 22% of the 10,621 patients with MDR-TB diagnosed in Maharashtra in 2019.^68^, ^69^, ^70^ Prescription of antibiotics prior to undergoing a TB test, and especially prescription of quinolones, carries the risk of delaying TB diagnoses by masking symptoms and thus delaying testing.^71^^,^^72^ Inappropriate use of quinolones is further associated with quinolone-resistance,^73^^,^^74^ and these are among the most widely used antibiotics in India.^75^ Our findings have potential implications for quality of TB care and, more generally, for antibiotic stewardship. It is essential to optimise antibiotic use in order to mitigate antibiotic resistance and infection transmission, and protect patient safety.^76^^,^^77^

Our study had several limitations to acknowledge. First, while the study was a population-weighted assessment of average practices among these provider types and cities, it was not necessarily representative of the provider mix that patients may face if they were to choose to visit different types of providers, and findings may not replicate in other settings. Patient choices in provider visitation might be influenced by factors like gender or socio-economic status, and these patient-level arbiters of care decisions could not be captured in this study design. Providers might choose to empirically treat poorer patients to save costs, but the SP methodology cannot account for this. Second, we did not directly inquire on rationale for empirical treatment and thus may not be able distinguish appropriate medicine use from misuse in a holistic sense. Third, observed practice only reflected what healthcare providers did when they came across a completely new patient seeking medical care during an initial visit. Although it was feasible in a pilot study to send back SPs for repeated interactions with the same provider,^17^ SPs have not yet been routinely used to construct standardized measures that include follow-up visits to providers. Fourth, this study only covered private practitioners in urban areas in India and cannot be extended to rural areas. In addition, SPs cannot measure the quality of care received over the entire treatment phase of TB care, because they are not in fact ill; therefore if any clinical indicators are assessed during any visit, physicians might act contrary to indication – even when a medical diagnostic result is provided (e.g. if the provider sees a healthy patient who lives in a polluted city with no cough, or fever or visible weight loss, they may ignore abnormal chest X-ray results). Moreover, since a proportion of SP-provider interactions resulted in direct dispensing of unlabelled medicines, certain drugs could not be identified, thus potentially leading to underestimation of the extent of antibiotic and other medicine dispensation. Furthermore, the intent of the overall study was not to inquire about this very particular question. As such, our design would surely have been strengthened if we had been able to have an exact counterfactual presentation of case 2. Specifically, we would have been able to expect direct comparability if we provided some SPs with a normal CXR. Next, it is possible that certain healthcare provider characteristics (such as years of experience (seniority), previous training on TB management) which we could not capture in this study, could potentially influence their decision to treat empirically. Finally, with the advent of the COVID-19 pandemic, the landscape of respiratory diseases worldwide has most likely changed since our study was conducted.^78^, ^79^, ^80^

This research contributes novel insights to the prevalence of empiric treatment based on CXR abnormality and providers’ behaviours associated with this practice. It stands as a reminder of the balance physicians in the Indian context need to find between not losing their patients altogether to follow-up and being able to trust their laboratories while providing the proper regimen and care to their patients. A more detailed inquiry into their practices and decision-making rationale, coupled with engaging them in antibiotic stewardship and lab strengthening measures, are likely to have impacts in timely and appropriate management of TB, and other respiratory infections.

## Contributors

Anita Svadzian - Data curation, Formal analysis, Methodology, Writing – original draft, Writing – review & editing, Benjamin Daniels - Project administration, Data curation, Methodology, Writing – review & editing, Giorgia Sulis - Data curation, Writing – review & editing, Jishnu Das - Conceptualization, Funding acquisition, Methodology, Supervision, Writing – review & editing, Amrita Daftary- Methodology, Supervision, Writing – review & editing, Ada Kwan- Data curation, Project administration, Methodology, Supervision, Writing – review & editing, Veena Das- Data curation, Methodology, Supervision, Writing – review & editing, Ranendra Das - Methodology, Supervision, Writing – review & editing, Madhukar Pai - Conceptualization, Funding acquisition, Methodology, Project administration, Supervision, Writing – review & editing.

## Data sharing statement

At the time of publication, data and analysis code will be made available at https://github.com/qutubproject/

## Declaration of interests

Madhukar Pai declares that he serves as an advisor to the following non-profit agencies in global health: 10.13039/100000865Bill & Melinda Gates Foundation; Foundation for Innovative New Diagnostics; 10.13039/100004423World Health Organization & the Stop TB Partnership. He declares no financial or industry conflicts. None of the other authors have any disclosures.

## Appendix A Supplementary data

Supplementary material

STROBE-checklist-v4-cross-sectional
