# Supplementary material for: Do private providers initiate anti-tuberculosis therapy on the basis of chest radiographs? A standardised patient study in urban India
Source: Lancet Reg Health Southeast Asia. 2023 Feb 2;13:100152. doi: 10.1016/j.lansea.2023.100152 (PMC10306035; doi:10.1016/j.lansea.2023.100152)

**Supplement: Do private providers initiate anti-tuberculosis therapy on the basis of chest radiographs? A standardised patient study in urban India**

Anita Svadzian PhD^1,2^, Benjamin Daniels MSc^3^, Giorgia Sulis MD PhD^4^, Jishnu Das PhD^3,5^, Amrita Daftary PhD^6,7^, Ada Kwan PhD^8^, Veena Das PhD^9^, Ranendra Das PhD^10^, Madhukar Pai MD PhD^1,2,11^

**Affiliations**

^1^ Department of Epidemiology, Biostatistics and Occupational Health, McGill University, Montreal, QC, Canada.

^2^ McGill International TB Centre, McGill University, Montreal, QC, Canada.

^3^ McCourt School of Public Policy, Georgetown University, Washington, DC, USA

^4^School of Epidemiology and Public Health, Faculty of Medicine, University of Ottawa, Ottawa, ON, Canada.

^5^ Centre for Policy Research, New Delhi, India

^6^ Dahdaleh Institute of Global Health Research, School of Global Health, York University, Toronto, ON, Canada

^7^ Centre for the Aids Programme of Research in South Africa MRC-HIV-TB Pathogenesis and Treatment

Research Unit, Durban, South Africa

^8^ Division of Pulmonary and Critical Care Medicine, Department of Medicine, University of California, San Francisco, California, USA

^9^ Department of Anthropology, Johns Hopkins University, Baltimore, USA

^10^ Institute for Socio-Economic Research on Development and Democracy, Delhi, India

^11^ Manipal McGill Program for Infectious Diseases, Manipal Centre for Infectious Diseases, Manipal Academy of Higher Education, Manipal, Karnataka, India.

**Corresponding author**:

Prof Madhukar Pai, MD, PhD

Dept. of Epidemiology, Biostatistics & Occupational Health

McGill University

2001 McGill College Avenue, Suite 1200

Montreal, QC, H3A 1G1

Email: [madhukar.pai@mcgill.ca](mailto:madhukar.pai@mcgill.ca)

Table of Contents

[S1-Text 1: SP Recruitment and Characteristics. 3](#_Toc111107821)

[S1-Text 2: Provider Sampling Framework. 4](#_Toc111107822)

[S1-Text 3: Weighting 5](#_Toc111107823)

[S1-Table 1: Standardised patient variable descriptions. 7](#_Toc111107824)

[S1-Table 2: Sampling and weighting distributions for cases 1 & 2 within formal providers surveyed. 9](#_Toc111107825)

[S1-Figure 1: Example of CXR image carried by female SPs. 12](#_Toc111107828)

[S1-Figure 2: Example of CXR image carried by male SPs. 15](#_Toc111107831)

[S1-Figure 3: Example of a prescription and subsequent, dispensed loose & unlabelled pills given to a case 2 presentation in Mumbai. 17](#_Toc111107833)

# **S1-Text 1: SP Recruitment and Characteristics.**

Methods of SP recruitment, script development, SP training, provider sampling and assignment of SP cases to providers was previously outlined by our team, and available as an open-access manual and toolkit.^15^ Briefly, the research team recruited staff from the local community and provides each SP with a single fixed script (example script in supplementary, S6-Figure 2) for a disease case scenario. These SPs are then extensively trained them to present discreetly at a large number of real healthcare practitioners, posing as a real care-seeker. Each provider’s actions and management approach was documented in a structured exit questionnaire (Appendix Y), completed within 1-2 hours of the scheduled visit with a sampled provider. Quality metrics were calculated and reported, including duration of the encounter, history questions asked, diagnoses given or suspected, laboratory tests ordered, treatments recommended/prescribed/dispensed, and price paid by the SP.

Over the course of the three rounds of data collection in each of the two cities, a total of 39 individuals (16 females and 23 males) were recruited and hired as SPs. A total of 13 of these individuals were hired as SPs in both cities, 17 were hired for Mumbai only and 9 for Patna alone.

In Mumbai, 30 individuals (11 female) conducted fieldwork between April 2015 and December 2019. SPs were originally from the states of Bihar (9), Madhya Pradesh (3), Delhi (2) and Maharashtra (16). Primary languages spoken by the SPs included Angika (1), Bengali (1), Bhojpuri (1), Hindi (9), Malwi (1), Magahi (4), and Marathi (12). In Patna, 22 individuals (10 female) conducted fieldwork. SPs were originally from the states of Bihar (18), Delhi (2) and Madya Pradesh (2). Primary languages spoken by the SPs included Angika (3), Bengali (1), Bhojpuri (4), Hindi (7), Maithili (1), Malwi (1) and Magahi (5).

All potential SPs underwent a health screening questionnaire and checkup, with the resulting cohort of actors of seemingly healthy status. This was important as it assured that the SPs had no apparent health conditions that could confound the case presentation and interaction with healthcare providers. While the SPs were specifically recruited to fit each case scenario and corresponding narrative, they differed in age, sex, height, and weight. The average age of all the SPs was 32. The youngest was 21 and the oldest was 58. The 23 males weighed 50 to 76 kilograms and were 1·55 to 1·84 meters tall. The 16 females weighed 46 to 73 kilograms and were 1·42 to 1·67 meters tall.

# **S1-Text 2: Provider Sampling Framework.**

The primary data collection for this study in Mumbai and Patna was conducted by urban TB programs funded by the BMGF and implemented by PPIAs in each city. These PPIAs were World Health Partners (WHP) in Patna and PATH in Mumbai. Each of these organizations were mapping, recruiting, and enrolling private sector providers into provider networks in both cities.^31^ Between 2015 and 2019, the QuTUB Project team completed three rounds of quality or care monitoring across the planned PPIA pilot cities of Mumbai and Patna, completing a total of 6,452 SP-provider interactions with primary care providers over the course of the study. A lane-by-lane mapping exercise^31^ resulted in a list of private sector providers in Mumbai and Patna, and this resulting universe was then restricted based on eligibility criteria for the SP study: providers eligible for the study were those who were known to see adult outpatients with respiratory symptoms in the private health sector. The description of the PPIA program served to support sampling weights applied to achieve the urban area estimates for Mumbai and Patna (described below).

Amongst formal providers across both cities, 701 providers were visited, resulting in a total of 4,166 SP interactions (case 1: 1,818, case 2: 795, case 3: 701, case 4: 852). In round 1 of data collection (2014-2015), 717 visits were made in Patna and 637 in Mumbai. In the following round (2016-2018), 761 visits were conducted in Patna vs. 635 interactions in Mumbai. The final round of visits (2019-2020) resulted in 674 and 742 visits amongst formal providers in Patna and Mumbai, respectively. In Patna, there were 963 case 1 presentations and 855 case 2 presentations. In Mumbai, SPs presented as case 1,390 times and as case 2, 405 times.

# **S1-Text 3: Weighting**

Based on the sampling strategy outlined above, the city-level estimates of the behavioural characteristics in Mumbai and Patna were extrapolates from the sampling frame to the full population of private healthcare providers in each city. To calculate averages and differences within and across cities, we utilized inverse probability weights to satisfy the following:

1. Each city-case combination (Patna case 1, Mumbai case 2, etc.) has a total sum of weights equal to one. Therefore, each case is equally weighted within each city, and the two cities have equal total weights.

2. Within each city-case combination, the sum of weights for (A) MBBS-qualified (formal) and above and (B) non-MBBS-qualified (informal) providers is exactly equal to each group’s prevalence in the city as a whole.

3. Within each city-case-qualification group, the relative total weights for (A) PPIA and (B) non-PPIA providers are exactly proportional to each group’s prevalence in that city and provider qualification stratum.

4. Each round of the study is exactly proportional to the number of visits completed within that round.

By satisfying these conditions, the weight on each interaction was calculated such that our estimates took the values that they would have had if we had sampled exactly at random from the city as a whole, assuming that our sample is representative of that provider mix. There are 96 weighting groups: one for each city, case, qualification, PPIA status, and round (2 * 4 * 2 * 2 * 3).

Under the assumption that the providers we sampled from our sampling frames were representative of similar providers throughout the city, the resulting estimates were thus representative of the choice of a random provider within the city for each case presentation. Thus, when a statistic is to be reported in this study in the form “X of Y interactions (N%)”, Y is the whole number of interactions observed, X is the whole number of interactions in which an outcome or characteristic occurred, and N is the population-level estimate calculated using the weights detailed above. Table 2-2 details the weights employed for case 2 presentations, by type of provider and city with S2-Table 2 in the Appendix detailing case 1 weighting as well.

# **S1-Text 4: Regression Model**

We presented both univariate and multivariate models for factors of interest, with the multiple regression framework specified as below (Equation 1):

*Equation 1:*

$$logit({Management}_{ijklt})= \alpha+ \beta_{0}+ \beta_{1}{Provider}_{jt}+ \beta_{3}{Patient}_{kt}+ \beta_{4}{Site}_{lt}+ \varepsilon_{ijklt}$$

where ${Management}_{ijklt}$ is one of the three empiric treatments by provider i to patient j at health facility k at time t; ${Provider}_{jt}$ is a vector of provider characteristics at time t; ${Patient}_{kt}$is a vector of patient characteristics at time t; ${Site}_{lt}$ is a vector of facility characteristics at time t; and ϵ is an error term. Relevant provider characteristics included patient yield; patient characteristics included disease demographic factors and clinical presentation; and facility characteristics included city. The final multivariate model only included variables that were shown to potentially impact care management outcomes in our previous work^11,15^ or would reasonably have contributed to care management practices.

# **S1-Table 1: Standardised patient variable descriptions.**

|  | Measurement method |
| --- | --- |
| Provider level- variables |  |
| Qualification of provider | Recorded in provider data |
| Provider younger than 30 years of age | Assessed by standardized patient |
| Provider 30–50 years of age | Assessed by standardized patient |
| Provider older than 50 years of age | Assessed by standardized patient |
| Provider male | Observed by standardized patient |
| Patients waiting on arrival | Observed by standardized patient |
| Patients waiting on departure | Observed by standardized patient |
| Provider has clinic assistant | Observed by standardized patient |
| Process indicator |  |
| Provider used cell phone | Observed by standardized patient |
| Other people in room during interaction | Observed by standardized patient |
| Television on during interaction | Observed by standardized patient |
| Essential checklist % | Calculated from standardized patient data |
| Time with provider (min) | Measured by standardized patient |
| Did the provider create a private environment? | Assessed by standardized patient |
| Did the provider explain about your illness? | Assessed by standardized patient |
| Did the provider explain your treatment plan? | Assessed by standardized patient |
| Did you like this doctor? | Assessed by standardized patient |
| Would you go to this doctor again? | Assessed by standardized patient |
| Did the provider seem knowledgeable about your illness? | Assessed by standardized patient |
| Did the provider address your worries seriously? | Assessed by standardized patient |
| How would you rate the provider? (1–10) | Assessed by standardized patient |
| Quality outcome |  |
| Correct management | Calculated from standardized patient data using Indian NTP guidelines |
| Correct Treatment based on Mx Dx | Calculated from standardized patient |
| Referred case | Reported by standardized patient |
| Tuberculosis suspicion | Reported by standardized patient |
| Chest CXR | Reported by standardized patient |
| Sputum acid-fast bacillus | Reported by standardized patient |
| Xpert MTB/RIF MTB/RIF | Reported by standardized patient |
| Anti-tuberculosis treatment | Determined by analysis team |
| Quinolone | Determined by analysis team |
| Other antibiotic | Determined by analysis team |
| Steroids | Determined by analysis team |

# **S1-Table 2: Sampling and weighting distributions for cases 1 & 2 within formal providers surveyed.**

|  |  |  | Weighting Group | | | |
| --- | --- | --- | --- | --- | --- | --- |
| Round | SP Case |  | Patna Formal Non-PPIA | Patna Formal PPIA | Mumbai Formal Non-PPIA | Mumbai Formal PPIA |
| Baseline | Case 1 | n | 253 | 136 | 134 | 171 |
|  | - | weight | 0.00352532 | 0.0007948 | 0.00714301 | 0.00025051 |
|  | Case 2 | n | 70 | 28 | 69 | 53 |
|  | - | weight | 0.01274152 | 0.00386048 | 0.01387193 | 0.00080824 |
| Round 1 | Case 1 | n | 215 | 89 | 117 | 156 |
|  | - | weight | 0.0041484 | 0.00121453 | 0.00818088 | 0.0002746 |
|  | Case 2 | n | 112 | 45 | 76 | 60 |
|  | - | weight | 0.00796345 | 0.00240208 | 0.01259425 | 0.00071395 |
| Round 2 | Case 1 | n | 189 | 81 | 122 | 155 |
|  | - | weight | 0.00471908 | 0.00133449 | 0.0078456 | 0.00027637 |
|  | Case 2 | n | 94 | 41 | 90 | 57 |
|  | - | weight | 0.00948837 | 0.00263643 | 0.01063515 | 0.00075152 |

# S1-Figure 1: Example of CXR image carried by female SPs.


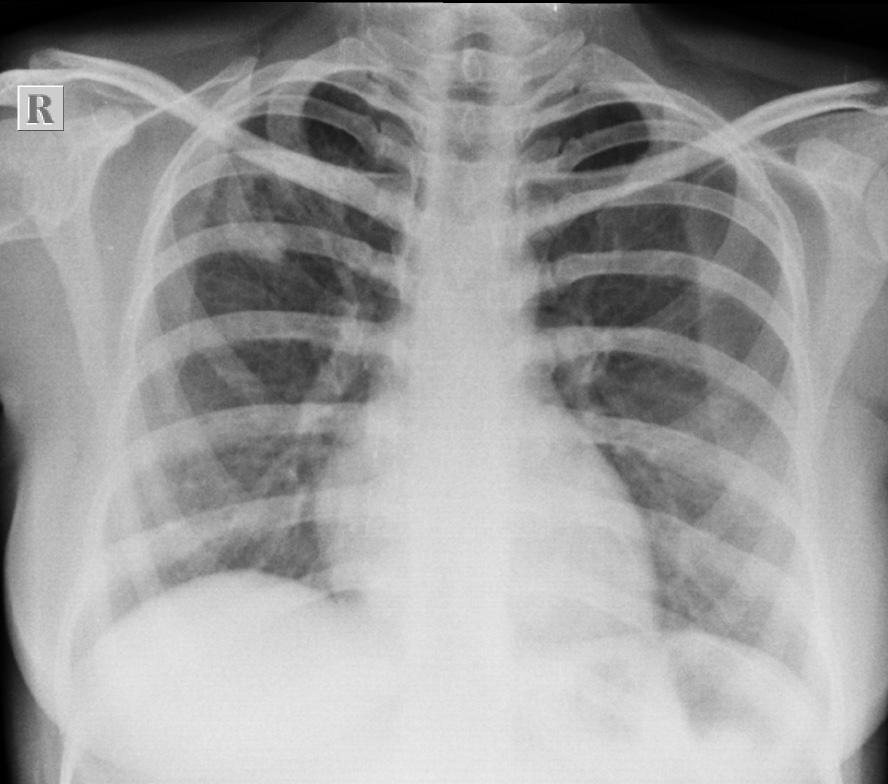


# S1-Figure 2: Example of CXR image carried by male SPs.


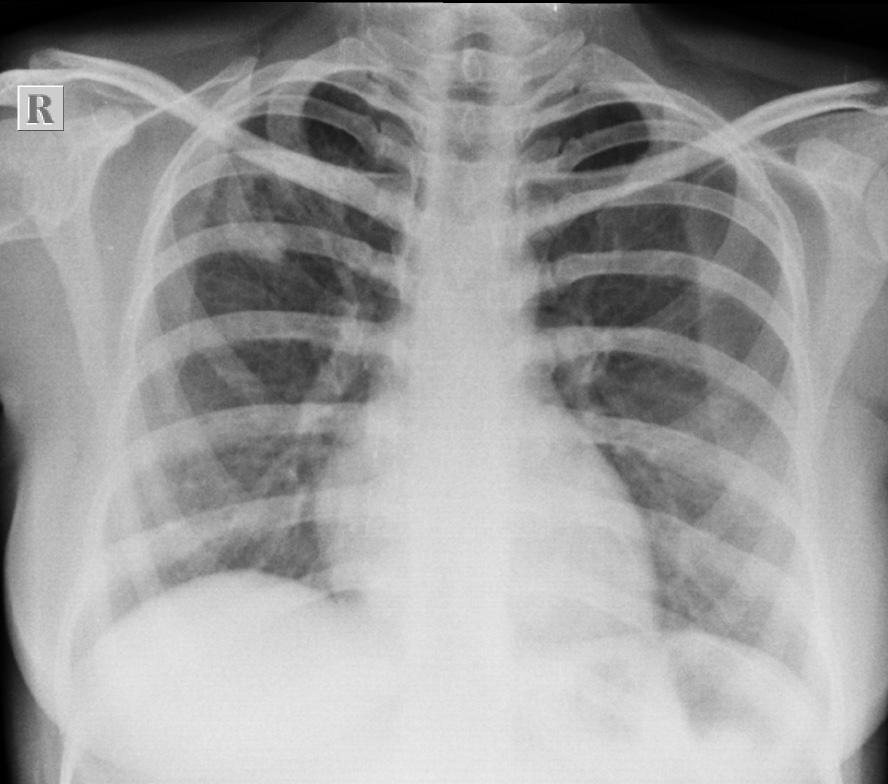


# S1-Figure 3: Example of a prescription and subsequent, dispensed loose & unlabelled pills given to a case 2 presentation in Mumbai.


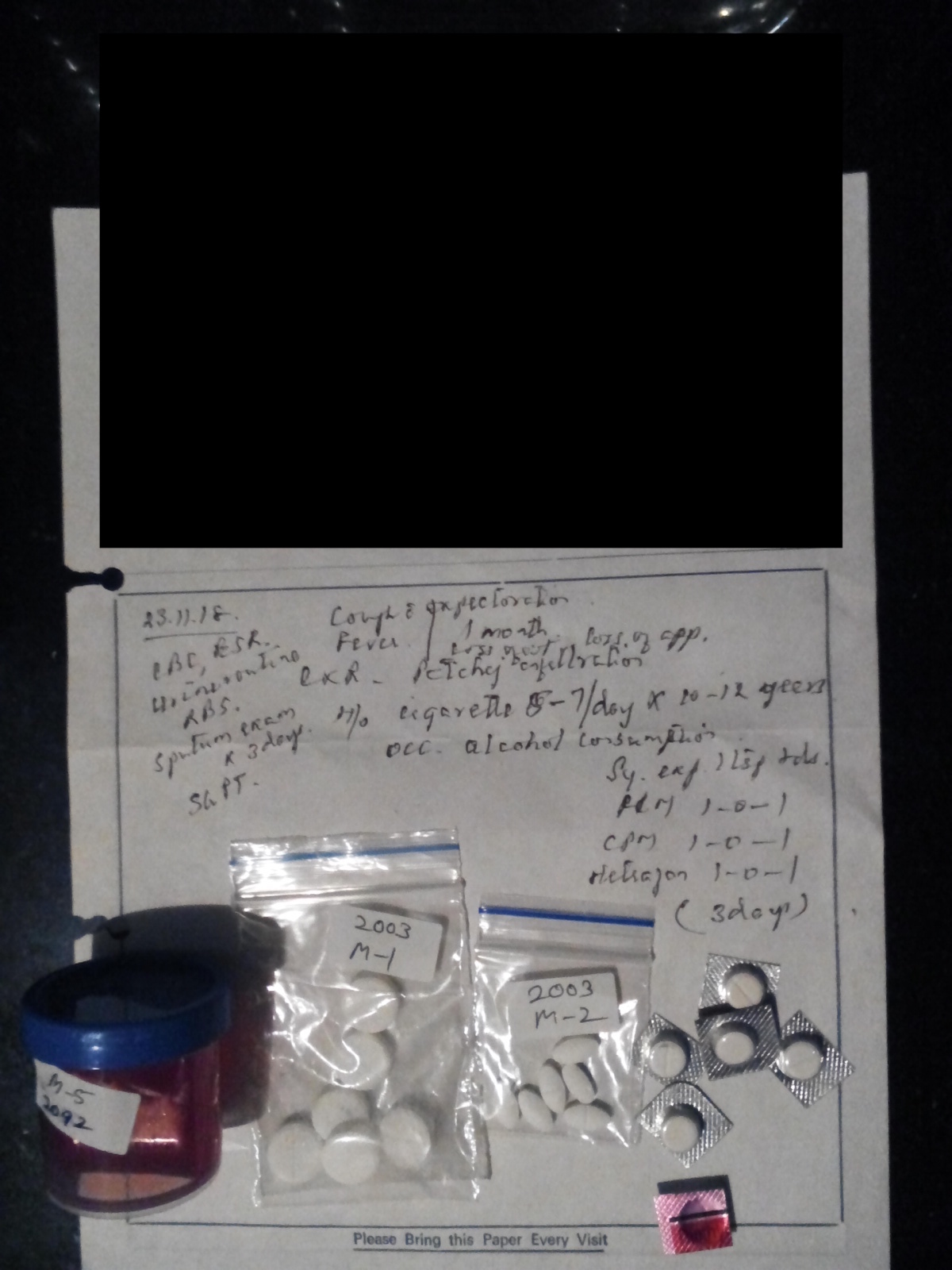

Supplement: Supplementary material [file mmc1.docx]
